# Enhancement of Y-bearing sperm enrichment in swamp buffalo using a TLR7/8 agonist (R848) combined with discontinuous Percoll density gradient centrifugation

**DOI:** 10.14202/vetworld.2026.1943-1953

**Published:** 2026-05-12

**Authors:** Naela Wanda Yusria Dalimunthe, Pakpoom Navanukraw, Saksiri Sirisathien, Sujira Thammawung, Patchara Phuektes, Panisara Kunkitti

**Affiliations:** 1 Veterinary Science Program, Faculty of Veterinary Medicine, Khon Kaen University, Khon Kaen, Thailand; 2 Veterinary Technology Study Program, Department of Bioresources Technology and Veterinary, Vocational College, Universitas Gadjah Mada, Yogyakarta, Indonesia; 3 Division of Theriogenology, Faculty of Veterinary Medicine, Khon Kaen University, Khon Kaen, Thailand; 4 Chiang Mai Artificial Insemination and Biotechnology Research Center, Chiang Mai 50300, Thailand; 5 Division of Pathobiology, Faculty of Veterinary Medicine, Khon Kaen University, Khon Kaen, Thailand

**Keywords:** buffalo breeding, density gradient centrifugation, frozen–thawed semen, R848, sperm motility, sperm sexing, swamp buffalo, Y-chromosome enrichment

## Abstract

**Background and Aim:**

Sex sorting of sperm is a valuable reproductive biotechnology for improving herd productivity and genetic management in livestock. Recent advances have explored Toll-like receptor 7/8 (TLR7/8) activation using resiquimod (R848) as a biological approach to selectively modulate sperm function. However, its application in swamp buffalo remains limited. This study aimed to evaluate the effectiveness of R848 combined with discontinuous Percoll density gradient centrifugation (PDGC) for enriching Y-bearing sperm in frozen–thawed swamp buffalo semen and to determine the optimal concentration to maximize enrichment without compromising sperm quality.

**Materials and Methods:**

Frozen–thawed semen from five swamp buffalo bulls (*Bubalus bubalis carabanensis*) was incubated in tris-based medium containing 0, 0.03, 0.3, and 1 μM R848 at 37°C for 45 min. Following incubation, samples were subjected to PDGC (45%/90%) to isolate motile sperm populations. The pellet fraction was reprocessed and evaluated for sperm kinematics using computer-assisted sperm analysis. The relative abundance of Y-bearing sperm was quantified by SYBR-based quantitative polymerase chain reaction targeting the *BRY4a* gene, normalized against *Glyceraldehyde*-3-phosphate dehydrogenase.

**Results:**

Quantitative polymerase chain reaction analysis demonstrated that treatment with 0.03 μM R848 resulted in a significant increase in BRY4a expression (approximately 2.6-fold) compared to other groups (p < 0.05), indicating enhanced Y-chromosome enrichment. Sperm motility parameters showed no significant differences in total and progressive motility between treated groups (0, 0.03, and 0.3 μM) and post-thawed controls (p > 0.05). However, velocity-related parameters such as average path velocity and curvilinear velocity increased significantly, while linearity decreased, reflecting a hyperactivation-like motility pattern. In contrast, the highest concentration (1 μM) negatively affected overall sperm motility and kinematic performance.

**Conclusion:**

The combination of low-dose R848 (0.03 μM) and PDGC effectively enhances the relative proportion of Y-bearing sperm in frozen–thawed swamp buffalo semen without detrimental effects on motility. This approach represents a cost-effective and field-applicable alternative to conventional sperm sexing methods, with potential applications in buffalo breeding and conservation programs. Further validation using larger sample sizes and fertility trials is recommended.

## INTRODUCTION

During the past decades, *Bubalus bubalis carabanensis (*swamp buffaloes) have played a crucial role in agriculture and food security across Southeast Asia, particularly in Thailand. Beyond their economic contributions, swamp buffaloes also hold significant cultural value, as reflected in traditions such as the Buffalo Racing Festival in Chonburi Province [1, 2]. Furthermore, the conservation and preservation of native buffalo genetics, including body size and morphology, are important considerations for breeders. Swamp buffalo populations have been declining due to reproductive challenges, including poor estrus expression, delayed puberty, a low number of primordial follicles, and overall low fertility. These factors significantly impact buffalo production and sustainability. To improve productivity and genetic selection, buffalo breeders increasingly adopt sperm sexing technologies to control offspring sex ratios, thereby reducing economic losses associated with undesired calves. In many swamp buffalo production systems in Thailand, male offspring are generally preferred for their larger body size, superior growth performance, and higher economic value for meat production and as breeding sires.

Spermatogenesis generates two distinct populations of spermatozoa, namely X- and Y-chromosome-bearing sperm, such that male gametes determine the sex of the offspring during fertilization. Several sperm sexing techniques have been developed, including density gradient centrifugation [3], immunological-based selection [4–6], magnetically activated cell sorting (MACS) [6], swim-up [7, 8], albumin column separation [9, 10], and flow cytometry [11, 12]. Among these technologies, flow cytometry remains the gold standard, achieving up to 90% purity in separating X- and Y-chromosome-bearing sperm based on DNA content [13, 14]. However, its implementation is constrained by the requirement for skilled personnel and sophisticated instrumentation [15, 16]. In addition, the use of semen sorted by flow cytometry has been associated with decreased reproductive performance, including a 23% reduction in pregnancy rate and a 24% reduction in calving rate [15]. Therefore, alternative cost-effective and field-applicable methods are needed.

Recent studies have explored Toll-like receptor 7/8 (TLR7/8) activation as a novel approach to sperm sexing. The TLR7/8 agonist R848 has been successfully applied in cattle for sperm sexing. Umehara *et al*. [17] reported that incubation with 0.03 μM R848 for 60 min significantly increased the proportion of XY embryos to 91.3 ± 2.8%, while also yielding 84.2 ± 5.3% XX embryos. Wen *et al*. [18] demonstrated that treatment with 0.6 μM R848 for 30 min resulted in Y-sperm enrichment of 88.6 ± 1.67% and X-sperm at 72.5 ± 2.12%. Huang *et al*. [19] achieved the highest Y-sperm enrichment (93.07 ± 1.26%) and embryo sex ratio (93.35 ± 1.10% XY embryos) using R848 combined with polyvinylpyrrolidone (PVP) for 50 min. These findings suggest that R848 is a promising method for sperm sexing in livestock.

Mechanistically, R848 selectively reduces X-sperm motility by impairing mitochondrial adenosine triphosphate (ATP) production, thereby creating motility differences that allow sperm separation [20]. This mechanism has been demonstrated in mice, cattle, goats, and sheep, where X-sperm exhibit reduced motility, facilitating preferential selection of Y-sperm [17–22]. However, the efficacy of R848-mediated sperm sexing appears to be species-dependent. A study in dogs [23] reported no significant enrichment following 0.4 μM R848 treatment for 60 min, with Y- and X-sperm proportions remaining comparable to controls. These findings indicate that species-specific variation in TLR7/8 signaling influences the effectiveness of this approach.

To enhance the separation efficiency, an additional physical selection step can be applied. Percoll-based density gradient centrifugation (PDGC) is widely used in bovine *in vitro* fertilization (IVF) due to its ability to selectively enrich motile sperm while removing dead cells and debris [24]. Its colloidal silica PVP composition enables effective separation based on sperm motility, maturity, and membrane integrity. PDGC has been shown to improve post-thaw sperm quality, reduce oxidative stress, and enhance fertilization and blastocyst development rates [24–31]. In R848-based sex selection, PDGC can be applied after incubation to physically separate sperm populations. Because sperm with higher motility preferentially migrate into the pellet fraction, this approach strengthens the differential effects induced by TLR7/8 activation and increases the likelihood of recovering Y-bearing sperm.

Despite advances in sperm sexing technologies, significant limitations remain in developing a practical, efficient, and species-specific method for swamp buffalo. Current gold-standard techniques, such as flow cytometry, are costly, technically demanding, and associated with reduced fertility outcomes, limiting their field applicability. Although TLR7/8 activation with R848 has shown promising results in several livestock species, its effectiveness is highly species-dependent, and its application in swamp buffalo remains underexplored. Furthermore, previous studies have primarily focused on either biochemical modulation or physical separation techniques, with limited investigation of their combined effects. The lack of integrated approaches that simultaneously exploit molecular signaling pathways and physical sperm selection mechanisms represents a critical knowledge gap. In addition, the optimal concentration of R848 for achieving effective Y-sperm enrichment without compromising sperm motility and functional integrity in swamp buffalo has not been clearly established. Therefore, there is a need for systematic evaluation of combined strategies that are both biologically effective and practically applicable under field conditions.

The present study aimed to evaluate the effectiveness of combining resiquimod (R848), a TLR7/8 agonist, with PDGC for sex selection of sperm in swamp buffalo. Specifically, this study sought to determine the optimal concentration of R848 that maximizes Y-bearing sperm enrichment while maintaining sperm motility and functional characteristics. In addition, the study aimed to assess whether the integration of molecular modulation through TLR7/8 activation and physical separation via PDGC could provide a cost-effective and field-applicable alternative to conventional sperm sexing techniques. The findings are expected to contribute to the development of practical strategies to improve reproductive efficiency, support genetic selection, and enhance the sustainability of swamp buffalo breeding and conservation programs.

## MATERIALS AND METHODS

### Ethical approval

This study was conducted in accordance with the ethical guidelines and regulatory standards for the use of animals in research. The experimental protocol was reviewed and approved by the Institutional Animal Care and Use Committee of Khon Kaen University, Thailand, under Approval Record No. IACUC-KKU (C)-112/67, Reference No. 660201.2.11/820 (60), dated October 25, 2024.

Frozen semen samples from mature swamp buffalo bulls were obtained from the Department of Livestock Development, Thailand. No live animals were handled, restrained, subjected to experimental procedures, or exposed to any intervention specifically for this study. Therefore, the study involved only the laboratory use of cryopreserved semen samples supplied by an authorized governmental source. All procedures involving semen handling, thawing, incubation, PDGC, sperm motility assessment, cryopreservation, DNA extraction, and quantitative polymerase chain reaction analysis were performed under approved laboratory conditions, in accordance with institutional biosafety and animal research guidelines.

### Study period and location

This study was conducted during 2024 at the Faculty of Veterinary Medicine, Khon Kaen University, Khon Kaen, Thailand. Laboratory analyses, including sperm processing and molecular evaluation, were performed in associated research laboratories equipped for reproductive biotechnology and molecular biology.

### Study design

Frozen semen from five mature swamp buffalo bulls, aged 4–12 years, was obtained from the Department of Livestock Development (Thailand). Frozen–thawed sperm were incubated with R848 at concentrations of 0, 0.03, 0.3, and 1 μM. Following incubation, sperm were subjected to PDGC to separate subpopulations based on physical and functional characteristics. Sperm collected from the pellet fraction were evaluated for motility using computer-assisted sperm analysis (CASA), while the remaining sperm were re-frozen for sex determination using real-time polymerase chain reaction (PCR).

Sperm sex was determined using real-time PCR with two primer sets: BRY4a, a Y-chromosome-specific marker, and GAPDH, used as an internal housekeeping gene control. Each sample was analyzed in triplicate.

### Reagent and media preparation

All reagents and chemicals were purchased from Sigma Chemical Company (St. Louis, MO, USA). The PDGC solution (Percoll™ Plus, Lot No. 10326907) was obtained from Cytiva (Uppsala, Sweden). Real-time PCR reagents were sourced from Qiagen (USA). Primers were supplied by Integrated DNA Technologies (IDT, USA).

### Semen preparation and sperm incubation

Sixteen frozen sperm straws cryopreserved in a 20% egg yolk–Tris (EYT) extender consisting of 250 mmol/L Tris (Tris-hydroxymethyl aminomethane), 90 mmol/L citric acid, 70 mmol/L fructose, 100 IU/mL penicillin G, 100 μg/mL streptomycin, 20% (v/v) fresh hen egg yolk, and 7% (v/v) glycerol were thawed at 37°C for 30 s. Immediately after thawing, sperm motility characteristics were assessed using CASA.

Thawed semen was aliquoted to 1 mL (10 × 10^7^ spermatozoa) and resuspended in 2 mL of Tris-based buffer (Tris 15.4 g, citric acid 8.65 g, glucose 6.25 g) supplemented with R848 (TLR7/8 ligand; SML0196-10MG, Sigma-Aldrich, St. Louis, MO, USA) to obtain final concentrations of 0 μM (control), 0.03 μM, 0.3 μM, and 1 μM. The 0 μM group served as a process-matched control, whereas the post-thawed group represented the untreated baseline. Samples were incubated at 37°C for 45 min in a humidified atmosphere containing 5% CO_2_.

### Sperm selection through discontinuous PDGC

Sperm separation was performed using a modified Percoll density gradient method adapted [32]. A 90% (v/v) Percoll solution was prepared by combining Percoll™ Plus (Cytiva, Uppsala, Sweden) with 10× Tyrode’s Albumin Lactate Pyruvate–HEPES (TALP-HEPES) buffer (9:1 ratio). The TALP-HEPES buffer consisted of sodium chloride, potassium chloride, sodium dihydrogen phosphate, and HEPES, supplemented with calcium chloride, magnesium chloride, and lactic acid, with pH adjusted to 7.3. A 45% Percoll solution was prepared by diluting the 90% Percoll solution with TALP-HEPES (1:1 ratio).

After incubation, 3 mL of semen from each group was layered onto a discontinuous Percoll gradient (2 mL 45% over 2 mL 90%) in a 15 mL conical tube and centrifuged at 590 g for 10 min (Hermle Z 320 centrifuge, Hermle Labortechnik, Wehingen, Germany). The bottom pellet (~1 mL), expected to be enriched in Y-bearing spermatozoa, was recovered and washed in 5 mL TALP-HEPES by centrifugation at 330 g for 7 min. The pellet was resuspended in TALP to a final volume of 500 μL and loaded into 0.5 mL straws for refreezing using EYT extender.

### Sperm freezing and thawing procedure

Sperm suspensions were loaded into 0.5 mL plastic straws and equilibrated in liquid nitrogen vapor at approximately 10 cm above the liquid nitrogen surface for 10 min. Following vapor-phase equilibration, the straws were plunged directly into liquid nitrogen and stored in cryogenic containers until further use.

### Assessment of sperm motility using CASA

Sperm motility characteristics were assessed using CASA (CEROS II system, Hamilton Thorne, Beverly, MA, USA; software version 1.14 Rev.A). After thawing at 37°C for 30 s, 15 μL of semen was placed in Leja chamber slides (20 μm; SC 20-01-C, Leja Products B. V., Nieuw-Vennep, The Netherlands) and incubated at 37°C for 5 min before analysis.

Samples were analyzed using a Nikon 10× objective (Nikon, Japan) with calibration factors of 1.21 for both X and Y axes. Videos were recorded at 37°C with a frame capture speed of 60 Hz and 30 frames per field. A minimum of 200 spermatozoa were analyzed per sample.

Cell detection parameters were defined as follows: head size 10–50 μm², head brightness ≥170, elongation 1–100%, tail brightness ≥70, and minimum large object area 1000 μm². Progressive motility was defined as average path velocity (VAP) ≥50 μm/s and STR ≥80%. Slow sperm were defined as VAP ≥20 μm/s and VSL ≥30 μm/s, whereas static sperm were defined as VAP ≤4 μm/s and VSL ≤1 μm/s.

Motility parameters included curvilinear distance (DCL), straight-line distance (DSL), average path distance (DAP), curvilinear velocity (VCL), VAP, straight-line velocity (VSL), straightness (STR), linearity (LIN), wobble (WOB), beat cross frequency (BCF), and amplitude of lateral head displacement (ALH).

### Quantification of Y-chromosome marker by real-time PCR

DNA sample preparation and extraction: Four 0.5 mL straws were thawed at 37°C for 30 s. Samples were transferred to 1.5 mL microcentrifuge tubes and centrifuged at 6010 g for 1 min. The supernatant was discarded, retaining approximately 100 μL of sperm pellet.

Sperm lysis was performed using a buffer containing Tris-HCl (pH 8.0), EDTA, sodium chloride, SDS, Proteinase K, and dithiothreitol at 55°C for 2 h. Genomic DNA was extracted using the DNeasy® Blood & Tissue Kit (Qiagen, Hilden, Germany) according to the manufacturer’s instructions. DNA was eluted in 80 μL AE buffer. DNA concentration and purity were measured using a BioDrop Duo spectrophotometer (BioDrop Ltd., Cambridge, UK), and samples were stored at −80°C until analysis.

Quantitative real-time PCR assay: Quantitative PCR (qPCR) was performed to determine the relative abundance of Y-chromosome-specific DNA sequences. Each 20 μL reaction contained 2 μL template DNA, ThunderBird® Next SYBR® qPCR Mix (Toyobo Co., Ltd., Osaka, Japan), and 0.4 μM of each primer. The gene BRY4a was used as the target, and GAPDH as the endogenous reference gene (Table 1).

**Table 1:** Primers used for real-time polymerase chain reaction.

Primer	Sequence	Annealing temperature (°C)	Fragment length	Reference
BRY4a	F: 5’-CTCAGCAAAGCACACCAGAC-3’	56	300 bp	[7]
	R: 5’-GAACTTTCAAGCAGCTGAGGC-3’			
GAPDH	F: 5’-ACA CCC TCA AGA TTG TCA GCA A-3’	55–56	102 bp	[25]
	R: 5’-TCA TAA GTC CCT CCA CGA TGC-3’			

Reactions were conducted in triplicate using a CFX96 real-time PCR detection system (Bio-Rad Laboratories, Hercules, CA, USA). Negative controls without template DNA were included. The thermal cycling protocol consisted of an initial denaturation at 95°C for 2 min, followed by 40 cycles of 95°C for 5 s, 58°C for 30 s, and 72°C for 30 s, with a final extension at 65°C for 5 s.

Relative gene expression was calculated using the 2^−ΔΔCT method, normalized to GAPDH expression [7]. Melting curve analysis confirmed amplification specificity, with a single peak observed for both BRY4a and GAPDH.

### Statistical analysis

All data are expressed as mean ± standard error of the mean (SEM). Data were assessed for normality and homogeneity of variance before analysis. Mean relative fold change, motility, and kinematic parameters were analyzed using one-way analysis of variance, with bulls (n = 5) treated as blocks and R848 concentrations as fixed factors.

Post hoc comparisons were performed using Duncan’s multiple range test when significance was detected (p < 0.05). Statistical analyses were conducted using SPSS software (SPSS Inc., Chicago, IL, USA), and graphs were generated using GraphPad Prism Version 9.3.0 (www.graphpad.com).

## RESULTS

### Assessment of sperm sex by real-time PCR

Quantitative real-time PCR analysis was conducted to assess the relative fold changes in BRY4a expression in sperm recovered from the pellet fraction following R848 treatment. Untreated spermatozoa served as the calibrator sample. Data were normalized to the endogenous reference gene GAPDH, and relative quantification was calculated using the 2^−ΔΔCt method. In the untreated control, ΔCt was defined as zero, yielding a relative expression value of 1.00. For treated samples, the 2^−ΔΔCt value reflected the fold change in BRY4a expression relative to the control [7].

**Figure 1:**
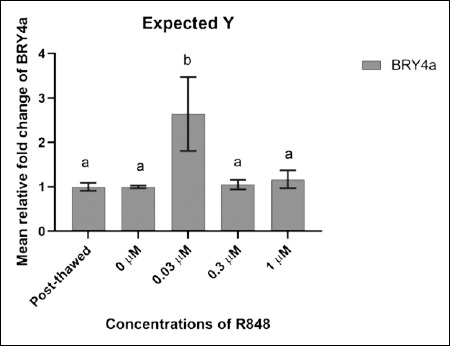
Relative expression (2^−ΔΔCt) of the Y-chromosome marker BRY4a in the pellet fraction following incubation with different concentrations of R848 and PDGC. Different superscript letters within the BRY4a column indicate statistically significant differences among treatments (p < 0.05).

The mean relative expression of BRY4a in semen treated with 0.03 μM R848 followed by PDGC showed a significantly higher value (p < 0.05) than the other groups (Table 2)., with an average 2^−ΔΔCt value corresponding to a 2.6-fold increase (Figure 1).

**Table 2:** Relative BRY4a expression (2^−ΔΔCt, mean ± SEM) in the pellet fraction following treatment with different concentrations of R848.

Concentration of R848	BRY4a (mean ± SEM)
Post-thawed	1.00 ± 0.23ᵃ
0 μM	1.00 ± 0.03ᵃ
0.03 μM	2.64 ± 0.83ᵇ
0.3 μM	0.88 ± 0.07ᵃ
1 μM	1.17 ± 0.20ᵃ

Values are expressed relative to the control and represent mean ± SEM. Different superscript letters within the BRY4a column indicate statistically significant differences among treatments (p < 0.05).

### Assessment of sperm motility using CASA

Sperm motility after thawing: After thawing, the mean percentages of total motile and progressive motile spermatozoa from five bulls were 79.0 ± 2.2% and 49.6 ± 5.3%, respectively. The VAP, VSL, and VCL were 92.0 ± 4.9 μm/s, 85.5 ± 4.9 μm/s, and 137.8 ± 5.4 μm/s, respectively. In addition, STR, LIN, and amplitude of lateral head displacement (ALH) were 93.0 ± 0.5%, 63.3 ± 1.7%, and 4.9 ± 0.4 μm, respectively.

Sperm motility after R848 treatment followed by PDGC: After treatment, sperm from the pellet fraction were resuspended and evaluated using CASA. The sperm motility patterns are presented in Table 3. Sperm recovery rates from the pellet fraction were 59.58 ± 15.67% (0 μM), 63.91 ± 13.17% (0.03 μM), 62.62 ± 9.64% (0.3 μM), and 56.76 ± 11.87% (1 μM).

**Table 3:** Motility characteristics of post-thawed buffalo sperm and post-treatment with different concentrations of R848 followed by Percoll density gradient centrifugation.

Treatment	R848 concentration	Motility (%)	Progressive (%)	VAP (μm/s)	VSL (μm/s)	VCL (μm/s)	STR (%)	LIN (%)	ALH (μm)	WOB (%)	BCF (Hz)
Post-thawed	–	78.98 ± 2.24ᵃ	49.64 ± 5.25ᵃ	92.03 ± 4.91ᵃ	85.52 ± 4.89ᵃ	137.77 ± 5.41ᵃ	92.97 ± 0.52ᵃ	63.30 ± 1.74ᶜ	4.90 ± 0.40ᵃ	67.85 ± 1.50ᶜᵈ	36.36 ± 2.33ᵇ
Post-treatment	0 μM	85.88 ± 2.04ᵃ	41.38 ± 3.09ᵃ	115.06 ± 5.45ᵇ	100.01 ± 5.99ᵃ	206.80 ± 14.41ᵇ	87.09 ± 3.06ᵃ	50.50 ± 3.10ᵃ	8.94 ± 0.82ᵇ	57.58 ± 2.17ᵃ	27.66 ± 1.72ᵃ
	0.03 μM	79.86 ± 5.00ᵃ	47.92 ± 3.50ᵃ	115.29 ± 5.90ᵇ	104.68 ± 5.11ᵃ	198.42 ± 11.22ᵇ	91.35 ± 0.54ᵃ	55.00 ± 1.40ᵃᵇ	8.39 ± 0.59ᵇ	60.04 ± 1.26ᵃᵇ	27.19 ± 0.77ᵃ
	0.3 μM	85.30 ± 1.19ᵃ	48.10 ± 2.51ᵃ	111.84 ± 6.67ᵇ	102.85 ± 5.93ᵃ	181.55 ± 13.45ᵇ	92.44 ± 0.19ᵃ	58.30 ± 1.53ᵇᶜ	7.71 ± 0.58ᵇ	63.09 ± 1.48ᵇᶜ	26.76 ± 0.81ᵃ
	1 μM	76.80 ± 1.75ᵃ	44.55 ± 1.68ᵃ	97.00 ± 6.72ᵃᵇ	90.55 ± 5.83ᵃ	142.83 ± 12.90ᵃ	93.57 ± 0.92ᵃ	65.18 ± 2.83ᶜ	5.78 ± 0.64ᵃ	69.49 ± 2.32ᵈ	29.16 ± 1.06ᵃ

VAP = Average path velocity, VCL = Curvilinear velocity, VSL = Straight-line velocity, STR = Straightness, LIN = Linearity, WOB = Wobble, ALH = Amplitude of lateral head displacement, BCF = Beat cross frequency. Different superscript letters within the same column indicate significant differences among treatment groups (p < 0.05). Data are presented as mean ± standard error of the mean (SEM) from three replications.

**Figure 2:**
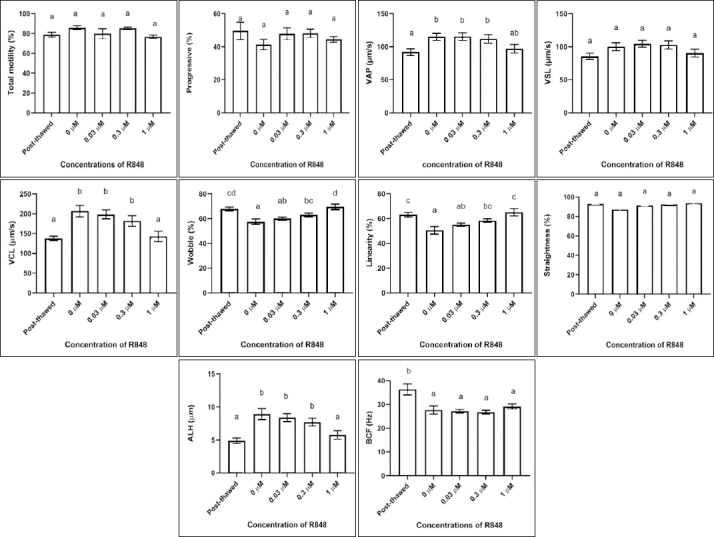
Motility characteristics of buffalo sperm post-thawed compared with post-treatment. Total motility = progressive motility, VAP = average path velocity, VSL = straight-line velocity, VCL = curvilinear velocity, STR = straightness, LIN = linearity (VSL/VCL × 100), ALH = amplitude of lateral head displacement, WOB = wobble (VAP/VCL × 100), BCF = beat cross frequency. Different letters above the bars indicate significant differences (p < 0.05).

There were no significant differences in total motility, progressive motility, VSL, and STR among treatment groups and the post-thawed group (p > 0.05). In contrast, significant increases in VAP, VCL, and ALH were observed after treatment (p < 0.05). Conversely, LIN, WOB, and BCF were significantly reduced (p < 0.05) (Table 3) (Figure 2).

## DISCUSSION

### Relative enrichment of Y-bearing sperm after R848 and PDGC treatment

The present study represents the first application of R848, a TLR7/8 agonist, combined with discontinuous PDGC in frozen–thawed swamp buffalo semen. The results suggest that this combined approach may increase the relative proportion of Y-bearing spermatozoa while maintaining motility parameters. Quantitative real-time PCR analysis showed that 0.03 μM R848 yielded the highest relative expression of the Y-chromosome marker BRY4a compared with the other groups. These findings indicate a relative enrichment of Y-bearing sperm at this concentration, as inferred from molecular quantification. However, the enrichment was assessed using relative qPCR analysis without absolute quantification or analytical validation of the BRY4a assay in swamp buffalo. Therefore, the findings should be interpreted as evidence of relative molecular enrichment rather than a precise determination of the proportion of Y-bearing sperm.

### Sperm motility and kinematic changes after treatment

Post-thaw motility characteristics confirmed that the semen used in this study exhibited high-quality motility, indicating that sperm maintained good functional integrity before experimental treatment. Following R848 treatment, spermatozoa presumed to be less responsive to TLR7/8 activation retained progressive motility and were preferentially enriched in the pellet fraction during PDGC. This observation is consistent with previous studies showing that spermatozoa with superior motility tend to migrate into denser layers under centrifugal force [24, 25, 30–35].

Comparisons between post-thawed and post-treatment sperm (0, 0.03, and 0.3 μM) revealed distinct changes in sperm kinematic patterns following treatment, incubation, and PDGC. Velocity parameters such as VCL and VAP increased significantly, while ALH was also elevated. In contrast, LIN decreased markedly. This combined profile, characterized by high VCL and ALH with low LIN, resembles kinematic patterns associated with hyperactivation, reflecting vigorous but less linear flagellar movement [36–38]. These motility patterns are biologically relevant, as previous studies have shown that R848-induced TLR7/8 activation suppresses ATP production and motility in X-bearing sperm, whereas Y-bearing sperm remain metabolically less affected and maintain stronger propulsive capacity [17–22, 39, 40]. Therefore, the increased motility parameters observed in this study likely reflect enrichment of highly motile Y-bearing sperm within the PDGC pellet fraction. Although the observed kinematic profile is consistent with hyperactivation, definitive confirmation of hyperactivation or capacitation would require additional functional or biochemical assays.

### Effect of high-dose R848 on sperm function

In contrast, exposure to the highest R848 concentration (1 μM) resulted in a markedly different outcome. CASA analysis showed significant reductions in total and progressive motility, as well as decreases in major velocity parameters (VCL, VAP, and VSL). Unlike the selective modulation observed at lower concentrations, 1 μM R848 appears to exert a broad inhibitory effect on overall sperm function. Excessive TLR7/8 activation at this concentration likely disrupts mitochondrial ATP synthesis and induces intracellular stress responses, impairing flagellar movement in both X- and Y-bearing spermatozoa [21]. These findings suggest that 1 μM exceeds the physiological tolerance threshold of frozen–thawed buffalo sperm, making it unsuitable for sperm sexing applications.

### Comparison with previous buffalo sperm sexing studies

The findings of the present study differ from those reported by Meel *et al*. [41], who achieved effective sex-specific separation in Murrah buffalo using 0.3 μM R848 during swim-up. Several factors may explain this discrepancy. First, differences in separation methods may contribute, as swim-up relies entirely on active upward movement of motile sperm, whereas PDGC incorporates additional physical stratification through density gradients and centrifugal force. This may allow motility differences between X- and Y-bearing sperm to manifest at lower R848 concentrations when PDGC is applied. Second, the study by Meel *et al*. [41] used fresh semen, whereas the present study used frozen–thawed semen. Cryopreservation can affect membrane integrity, receptor distribution, and metabolic responsiveness, potentially increasing sensitivity to R848 at lower concentrations. Third, differences in media composition and capacitation status may influence the molecular interaction between R848 and spermatozoa. Finally, cryodamage and reduced membrane stability in post-thaw sperm may facilitate differential ligand responsiveness, contributing to the observed concentration-dependent effects.

### Practical implications for buffalo breeding and conservation

In summary, integration of molecular evidence with kinematic data indicates that the use of a low-dose of R848 (0.03 μM) combined with PDGC may increase the proportion of Y-bearing spermatozoa in frozen–thawed swamp buffalo semen while preserving motility parameters. This approach may provide a practical and cost-effective strategy applicable to semen stored in genetic resource banks. The technique has potential implications for conservation and breeding programs aimed at improving and managing swamp buffalo populations in Thailand. Increasing the proportion of male offspring may support agricultural labor demands and contribute to long-term sustainability of buffalo genetic resources. However, further validation in larger populations and through functional fertility assessments is necessary before routine application can be recommended.

## CONCLUSION

The present study demonstrated that the application of a low concentration of R848 (0.03 μM) combined with PDGC can increase the relative abundance of Y-bearing spermatozoa in frozen–thawed swamp buffalo semen without compromising sperm motility. Molecular evaluation using qPCR targeting BRY4a revealed a significant increase in relative expression at 0.03 μM compared with other treatment groups (p < 0.05), indicating effective enrichment of Y-chromosome-bearing sperm. In parallel, CASA-based kinematic analysis showed that key motility parameters such as VAP, VCL, and ALH increased, while LIN decreased, suggesting a shift toward hyperactivation-like motility patterns that may favor the selection of highly motile Y-bearing sperm.

From a practical perspective, this combined approach provides a promising alternative to conventional sperm sexing techniques such as flow cytometry, which are often limited by high cost, technical complexity, and reduced fertility outcomes. The integration of molecular modulation through TLR7/8 activation and physical separation via PDGC offers a relatively simple, cost-effective, and field-applicable method. This strategy is particularly relevant for buffalo production systems in Thailand and similar regions, where increasing the proportion of male offspring can enhance meat production, improve breeding efficiency, and support agricultural labor demands.

A key strength of this study lies in the combined evaluation of molecular and functional parameters, linking BRY4a-based relative quantification with detailed CASA-derived kinematic analysis. This integrative approach provides a more comprehensive understanding of sperm selection dynamics compared with studies relying on a single method. In addition, the use of frozen–thawed semen enhances the practical applicability of the findings, as it reflects real-world conditions in artificial insemination and genetic resource banking systems.

However, several limitations should be acknowledged. The study relied on relative qPCR quantification without absolute validation of Y-bearing sperm proportions, thereby limiting the precise interpretation of enrichment efficiency. The sample size was relatively small, with semen obtained from only five bulls, and inter-individual variability in response to R848 treatment was not assessed. Furthermore, functional fertility outcomes, such as fertilization rates and offspring sex ratios *in vivo*, were not evaluated.

Future research should focus on validating these findings using larger and more diverse buffalo populations, incorporating absolute quantification techniques and advanced molecular assays. Studies assessing fertilization outcomes *in vitro* and *in vivo*, embryo development, and offspring sex ratios are essential to confirm the biological effectiveness of this approach. In addition, optimization of incubation conditions, interaction with different semen extenders, and long-term effects on sperm viability and DNA integrity should be investigated.

In conclusion, the combined use of R848 at an optimized low concentration and PDGC represents a promising and practical strategy for enhancing enrichment of Y-bearing sperm in swamp buffalo semen. While the current findings provide strong preliminary evidence, further validation through functional and large-scale studies is required before routine application in breeding programs. This approach has the potential to contribute significantly to sustainable buffalo production, genetic improvement, and conservation efforts.

## DATA AVAILABILITY

All data generated or analyzed during this study are included in this published article.

## AUTHORS’ CONTRIBUTIONS

PK: Conceptualization, methodology, investigation, writing – review and editing, and supervision. NWYD: Conceptualization, methodology, investigation, and writing – original draft. PN: Methodology, investigation, and writing – review and editing. SS: Conceptualization, methodology, investigation, writing – review and editing, and supervision. ST: Sample collection and writing – review and editing. PP: Methodology and writing – review and editing. All authors have read and approved the final version of the manuscript.
